# Characterization of the complete chloroplast genome of *Scutellaria amoena* C. H. Wright (Lamiaceae), a medicinal plant in southwest China

**DOI:** 10.1080/23802359.2019.1666677

**Published:** 2019-09-18

**Authors:** Qi Chen, Dequan Zhang

**Affiliations:** a College of Pharmacy and Chemistry, Dali University, Dali, China;; b Institute of Materia Medica, Dali University, Dali, China;; c Key Laboratory of Yunnan Provincial Higher Education Institutions for Development of Yunnan Daodi Medicinal Materials Resources, Yunnan, China

**Keywords:** *Scutellaria amoena*, medicinal plant, complete chloroplast genome, phylogeny, Scutellariae Radix

## Abstract

*Scutellaria amoena* is a frequently-used medicinal plant in Southwerst China. In this study, we sequenced the complete chloroplast (cp) genome sequence of *S. amoena* C. H. Wright to investigate its phylogenetic relationship in the family Lamiaceae. The chloroplast genome of *S. amoena* was 151, 569 bp in length with 38.4% overall GC content, including a large single copy (LSC) region of 83,739 bp, a small single copy (SSC) region of 17,326 bp and a pair of inverted repeats (IRs) of 25,252 bp. The cp genome contained 112 genes, including 79 protein-coding genes, 29 tRNA genes, and 4 rRNA genes. The phylogenetic analysis indicated *S. amoena* was closely related to *S. baicalensis* that is used as original species of Scutellariae Radix.

*Scutellaria* L. is a large genus of the Lamiaceae family, which includes 350 species all over the world (Willis 1966). Most of the species are widespread in temperate regions and tropical mountains of Europe, North America and East Asia (Bruno et al. 2002). There are 98 species in China (Li and Ian 1994). Some species in this genus have been widely used in traditional Chinese medicine for thousands of years (Jiangsu New Medical College 1977). Among these species, *S. baicalensis* Georgi is an original species of famous traditional Chinese medicine, namely Scutellariae Radix (National Pharmacopoeia Committee 2015). In southwest China, *S. amoena* C. H. Wright is used as the alternative of genuine medicine for the treatment of flammation, allergy, diarrhea, bronchitis and hepatitis (Zhang et al. 1998). However, until now, most of the studies for this species mainly focused on describing its chemical compositions (Hu and Liu 1989; Zhou et al. 1999; Shang et al. 2010) and quantitative analysis of high performance liquid chromatography (HPLC) methods (Zhang et al. 1998), with little involvement in its molecular biology. Here, we reported the complete chloroplast genome sequence of *S. amoena* and revealed its phylogenetic relationships with other species in the Lamiaceae.

Fresh and clean leave materials of *S. amoena* were sampled from Eryuan county, Yunnan, China (N25.84°, E100.09°). Meanwhile, a voucher specimen (No. ZDQ17067) was collected and deposited at the Herbarium of Medicinal Plants and Crude Drugs of the College of Pharmacy and Chemistry, Dali University. The total genomic DNA was extracted using the improved CTAB method (Doyle 1987; Yang et al. 2014), and sequenced with Illumina Hiseq 2500 (Novogene, Tianjing, China) platform with pair-end (2 × 300 bp) library. About 3.46 Gb of raw reads with 11,540,174 paired-end reads were obtained from high-throughput sequencing. The raw data were filtered using Trimmomatic v.0.32 with default settings (Bolger et al. 2014). Then paired-end reads of clean data were assembled into circular contigs using GetOrganelle.py (Jin et al. 2018) with *Pogostemon yatabeanus* (No. NC_031433) as a reference. Finally, the cpDNA was annotated by the Dual Organellar Genome Annotator (DOGMA; http://dogma.ccbb.utexas.edu/) (Wyman et al. 2004) and tRNAscan-SE (Lowe and Chan 2016).

The annotated chloroplast genome was submitted to the GenBank under the accession number xxx. The total length of the chloroplast genome was 151,569 bp, with 38.4% overall GC content. With typical quadripartite structure, a pair of IRs (inverted repeats) of 25,252 bp was separated by a small single copy (SSC) region of 17,326 bp and a large single copy (LSC) region of 83,739 bp. The cp genome contained 112 genes, including 79 protein-coding genes, 29 tRNA genes, and 4 rRNA genes. Of these, 18 genes were duplicated in the inverted repeat regions, 12 genes, and 6 tRNA genes contain one intron, while two genes (*ycf3* and *clpP*) have two introns.

To investigate its taxonomic status, a total of 24 cp genome sequences of Lamiaceae species were downloaded from the NCBI database used for phylogenetic analysis. After using MAFFT V.7.149 for aligning (Katoh and Standley 2013), jModelTest v.2.1.7 (Darriba et al. 2012) was used to determine the best-fitting model for the chloroplast genomes. Then Bayesian inference (BI) was performed by MrBayes v.3.2.6 (Ronquist et al. 2012) with *Tanaecium tetragonolobum* (No. NC_027955) as outgroup. The results showed that *S. amoena* was closely related to *S. baicalensis* (Figure 1). This indicated that using roots of *S. amoena* as the alternative of Scutellariae Radix was reasonable in phylogeny. Moreover, the genus *Scutellaria* L. possessed close phylogenetic relationships with *Pogostemon* Desf. and *Ajuga* L. The present study afforded scientific evidence for resource development of Scutellariae Radix and would be beneficial to taxonomy and phylogeny of Lamiaceae.

**Figure 1.**
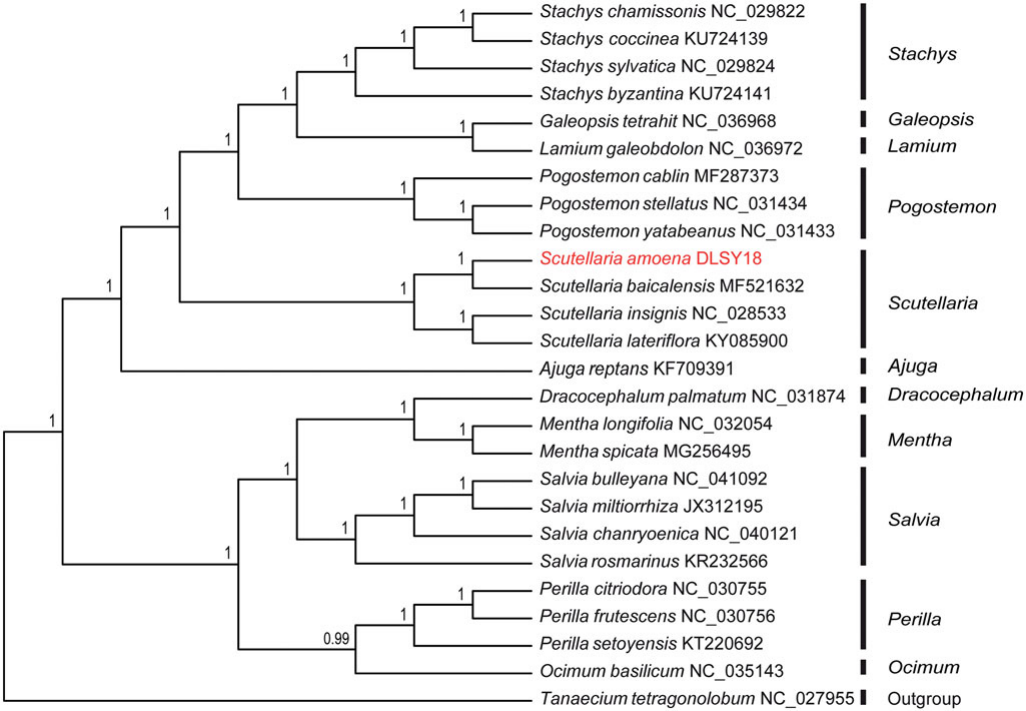
Bayesian inference (BI) tree of 25 species in the family Lamiaceae based on the complete chloroplast sequences using *Tanaecium tetragonolobum* (No. NC_027955) as an outgroup.
